# Rheumatic Chorea as the First Presenting Sign in a 13-year-old Female Child

**DOI:** 10.7759/cureus.5447

**Published:** 2019-08-21

**Authors:** Alina Sehar, Saad Nasir, Arshi Seja

**Affiliations:** 1 Internal Medicine, United Medical and Dental College, Karachi, PAK; 2 Internal Medicine, United Medical and Dental College/Creek General Hospital, Karachi, PAK; 3 Pediatrics, United Medical and Dental College/Creek General Hospital, Karachi, PAK

**Keywords:** rheumatic fever, sydenham chorea, case report, rheumatic chorea, pediatrics, group a beta-hemolytic streptococcus infection

## Abstract

Rheumatic chorea (RC) is a movement disorder seen in young children and adolescents with a recent history of incompletely treated group A beta-hemolytic streptococcal (GABHS) pharyngitis. Although, it rarely presents as the first manifestation of the disease, physicians should be aware of the disease, so that early diagnosis and prompt treatment may lead to elimination of the pathogen and prevent further disease progression. We present a case of a 13-year-old female child who presented with only RC as the first clinical sign.

## Introduction

Rheumatic chorea (RC) is a debilitating neurological presentation of rheumatic fever (RF). RC is a rare isolated and initial presentation of RF [1]; however, with rheumatic carditis it becomes fairly common [2]. Chorea alone has been identified in 0.6% cases of RF in Nepal and along with carditis its incidence increased to 2.3% [3]. In Pakistan, 16% children with rheumatic carditis developed chorea in the first presentation and another 4% in recurrent attacks [2]. RF occurs as the autoimmune response of the body to group A beta-hemolytic streptococcal (GABHS) infections. These infections are more common in school-going children and arthritis and carditis remain the common presentation in this age group. RC remains the most common cause of any choreiform movement in children. Failure to recognize chorea as a presenting sign of acute RF and subsequent management predisposes the child to recurrent attacks of RF and also rheumatic heart disease [4]. We present a case of chorea as the first presentation of RF in a 13-year-old child.

## Case presentation

A 13-year-old female child was brought by her mother to the outpatient department (OPD) of a tertiary care hospital, in Karachi, with a two-day history of sudden onset of restless abnormal movements of the body. The abnormal movements first began bilaterally in the upper limbs then involved the trunk (on the same day) and finally both lower limbs were involved (on day 2). Her mother reported she has difficulty performing her daily activities such as bathing, picking up utensils, and eating since then. The mother also reported that there were no abnormal, involuntary movements during sleep. She had a history of sore throat and fever three weeks back, which resolved spontaneously.

The general physical examination was unremarkable. She was alert and oriented to place and time. While lying, she was anxious and restless with involuntary movements. Her vitals were taken which showed, heart rate 92/min, respiratory rate 22/min, afebrile, and blood pressure 105/80 mmHg. She was mildly anemic. Pertinent examination findings included unclear speech, difficulty in walking, and generalized hypotonia. There were irregular contractions and relaxation of muscles showing pronation of forearm on outstretched hand above her head called as “Milkmaid's sign.”

Her laboratory investigations showed hemoglobin (Hb) levels: 11.7 mg/dL (normal range: 12.0-15.5), hematocrit (HCT) level: 40.5% (normal range: 37%-48%), total leukocyte count (TLC): 7600 IU/L (normal range: 130,000-400,000), erythrocyte sedimentation rate (ESR): 55 mm 1st hour (normal: <20), antistreptolysin O (ASO titer): 584 (normal: 166 Todd units), electrocardiogram (ECG) and echocardiogram were with normal PR interval of 0.12 s thus; rheumatic carditis was ruled out.

The patient was initially managed with intravenous (IV) fluids, IV ceftriaxone, and oral phenobarbitone. After 48 h of hospital admission, she was started with oral aspirin along with antacids and haloperidol. Joint pain and swelling was observed within the next 48 h and the patient was discharged on aspirin 70 mg/kg/day for five days followed by 50 mg/kg/day for three weeks. Oral phenobarbitone and haloperidol were also advised for 15 days. Injection Benz penicillin 1.2 mil IU every three weeks (till the age of 21) was also advised. At her first follow-up visit three weeks later, child’s condition had significantly improved and the dose of aspirin was tapered with 25 mg/kg/day for the next two weeks. The patient has since resumed her normal daily routine activities with resumption of her school and continues to receive injection Benz penicillin every three weeks. 

## Discussion

Rheumatic chorea or Sydenham chorea (SC) is a neurological disorder of childhood triggered as a result of autoimmune response to GABHS infections. SC is categorized as a major criterion for acute RF diagnosis. As per modified Jones criteria, clinical appreciation of choreiform movements is sufficient to diagnose RF in the presence of acute infective history [5-6]. Females develop choreiform movements more commonly than males [3]. It is characterized by sudden, brief, nonrhythmic, nonrepetitive twitching of limbs with facial grimacing. There is a history of sore throat several weeks before onset of symptoms which include, involuntary movements, slurred speech, hypotonia, and difficulty holding objects, writing, eating, and dressing [6].

To the best of our knowledge, this is the first report of pure RC in a child from Pakistan. Other pediatric reports have been in presence of rheumatic carditis as discussed earlier [2], however, in our case, cardiac complications were rules out on echocardiogram. In a recent Pakistani prospective study with 60 acute AF children, no case of chorea was appreciated either as the first episode or even as recurrences [7]. There have been other case reports of pediatric RF-both pure as well as with carditis-from other regions including Nepal (13-year-old female) [8], Saudi Arabia (5-year-old male) [4], India (18-year-old female) [5], and the United States (7-year-old female) [9].

In a four-year retrospective analysis from Nepal, 672 cases of pediatric acute RF were identified [3]. There were more females than males (55% vs. 45%). The incidence of SC was 3.8%. SC was much more common in females than males (77% vs. 23%) and 73% lied between the age of 10 and 16 years. Only 0.6% children had isolated chorea, 0.8% had chorea with arthritis, and 2.3% had chorea with carditis [3]. In an Israeli study with 24 SC patients, there were twice as many female children as male. Their ages ranged from 4 to 13 years. Familial chorea was appreciated in 8.3%, 21% had pure isolated chorea, and 42% developed recurrent choreic episodes in the subsequent years [10].

## Conclusions

Pure isolated chorea as the first presentation of rheumatic fever cannot be underestimated in older children. RC should be among the top differential diagnoses in children presenting with movement disorders in developing countries. Keen observation of the progression of erratic movements helps in suspecting chorea. With strong clinical suspicion unnecessary neurological investigations can be avoided. Recognition of RC and its timely management is crucial in preventing rheumatic heart disease.

## References

[1] Ilgenfritz S, Dowlatshahi C, Salkind A (2013). Acute rheumatic fever: case report and review for emergency physicians. J Emerg Med.

[2] Chagani HS, Aziz K (2003). Clinical profile of acute rheumatic fever in Pakistan. Cardiol Young.

[3] Regmi PR, Shrestha A, Khanal HH, Nepal BP, Chapagain P (2012). Prevalence of Sydenham’s chorea in patients with acute rheumatic fever in Nepal. NHJ.

[4] Lardhi AA (2014). Sydenham chorea in a 5-year-old Saudi patient. Neurosciences.

[5] Manjunath C, Vinay HR, Das K, Ghorpade VA (2018). An atypical case of rheumatic chorea in a rural tertiary health centre of South India. Int J Contemp Pediatr.

[6] Walker KG, Wilmshurst JM (2010). An update on the treatment of Sydenham's chorea: the evidence for established and evolving interventions. Ther Adv Neurol Disord.

[7] Sheikh AM, Sadiq M, Rehman AU (2016). Changing clinical profile of acute rheumatic fever and rheumatic recurrence. J Ayub Med Coll Abbottabad.

[8] Joshi A, Shrestha RPB, Shrestha PS, Dangol S, Shrestha NC, Poudyal P, Shrestha A (2015). Sydenham’s chorea as presentation of rheumatic heart disease. Kathmandu Univ Med J.

[9] Chandnani HK, Jain R, Patamasucon P (2015). Group C Streptococcus causing rheumatic heart disease in a child. J Emerg Med.

[10] Korn-Lubetzki I, Brand A, Steiner I (2004). Recurrence of Sydenham chorea: implications for pathogenesis. Arch Neurol.

